# Mood, Behavioral Impairment, and Sleep Breathing Disorders in Obstructive Sleep Apnea Syndrome Patients Treated with Maxillomandibular Advancement: Reflection on a Case Series and Review of Literature

**DOI:** 10.3390/jpm13101425

**Published:** 2023-09-22

**Authors:** Giovanna Stilo, Carmelo Lo Faro, Isabella Pollicina, Loredana Falcone, Paola Campagna, Silvia Felis, Salvatore Crimi, Ignazio La Mantia, Rosalia Leonardi, Alberto Bianchi

**Affiliations:** 1 Department of Medical and Surgical Sciences and Advanced Technologies “GF Ingrassia”, ENT Section, University of Catania, 95100 Catania, Italy; giovastilo@gmail.com (G.S.); isabellapollicina90@gmail.com (I.P.); silvia.felis@outlook.com (S.F.); igolama@gmail.com (I.L.M.); 2 Department of General Surgery and Surgical-Medical Specialties, School of Dentistry, University of Catania, 95124 Catania, Italy; loredanastellafalcone@gmail.com (L.F.); paolacampagna91@gmail.com (P.C.); 3 Department of General Surgery, Section of Maxillo Facial Surgery of Polyclinic “G. Rodolico-San Marco” University Hospital, University of Catania, 95100 Catania, Italy; torecrimi@gmail.com (S.C.); rleonard@unict.it (R.L.); alberto.bianchi@unict.it (A.B.)

**Keywords:** obstructive sleep apnea syndrome, cognitive symptoms, depression, BDI

## Abstract

The correlation between depressive and cognitive symptoms and OSAS (obstructive sleep apnea syndrome) is between 5 and 63%. We reported the case of two patients with severe OSAS and its associated depressive symptoms that were intolerant to continuous positive airway pressure (C-PAP) and underwent maxillomandibular advancement (MMA) surgery. The severity of cognitive and depressive symptoms was assessed using validated questionnaires (Beck Depression Inventory, Beck Anxiety Inventory, Epworth Sleepiness Scale, and quality of life), medical observation, and patient-reported symptoms. We performed pre- and post-treatment polysomnography. Six months after treatment, the value of the apnea–hypopnea index (AHI) had returned to the normal range and, together with it, the depressive component was considerably reduced and the patients’ overall quality of life (BDI, BAI, ESS, and qol) improved. Conclusion: We described significant improvement in all the analyzed parameters, such as physical and mental functioning, and depression and anxiety rates.

## 1. Introduction

Obstructive sleep apnea syndrome (OSAS) is a respiratory sleep disorder characterized by recurrent episodes of partial or complete upper airway collapse during the night, leading to apnea and hypopnea events, intermittent hypoxia, oxygen desaturations, and arousals [1,2]. Sleep fragmentation and intermittent hypoxia lead to daytime sleepiness, difficulty concentrating, and behavioral and mood disorders, such as depression and anxiety [3].

Respiratory disorders in sleep are attributable to alterations in the mechanical ventilation that are of complex and often multidisciplinary origin. The most common cause of such disorders is obstructive sleep apnea syndrome, a condition characterized by repeated partial or complete obstructions of the upper airways during sleep, which generally occur at the level of the hypopharynx and/or at the retropalatal level resulting in a multilevel obstruction [2]. They can cause episodes of apnea or hypopnea, resulting in intermittent arterial oxygen desaturation and sleep fragmentation. The American Academy of Sleep Medicine’s guidelines classify the severity of OSAS with the Apnea–Hypopnea index (AHI). The gold standard for the treatment of OSAS is continuous positive pressure ventilation (C-PAP), which is useful for the correction of obstructive episodes in the upper airways and the resulting hypoxemia during sleep [3]. Frequent micro-awakenings cause wake-up headache, memory impairment, irritability, and daytime sleepiness. At the cardiovascular level can be observed an increase in systemic and pulmonary blood pressure, post-load of the left ventricle and heart rate, with the possible appearance of arrhythmias (atrial fibrillation, bradyarrhythmias, and ventricular and supraventricular arrhythmias). OSAS is considered an independent risk factor for coronary heart disease and cerebrovascular events.

Executive functions, which are the most frequently compromised, include programming, attention building, and short-term verbal/nonverbal memory [4,5,6,7,8,9]. Liu X et al. reported that intermittent hypoxia (IH) conditions in OSAS patients resulted in neuronal lesions (especially in the hippocampus and cortex), leading to cognitive dysfunction, a significant and extraordinary complication in OSAS patients [10].

It is estimated that about one billion of the world’s population between 30 and 70 years old is affected by OSAS, and about half of this (425 million) needs treatment [5]. The gold-standard treatment of moderate/severe OSAS currently is positive pressure pulmonary ventilation with C-PAP (continuous positive airways pressure), which reduces comorbidities, including long-term cardiovascular risk, and symptoms related to OSAS, but these effects are determined by adherence to therapy [8]. Adherence to therapy with C-PAP is a multifactorial phenomenon that can be improved through educational and informative paths on the consequences of OSAS and the benefits of C-PAP. Literature data tell us, however, that only 40–60% of patients use C-PAP as prescribed, which is why a good portion of these patients are likely to be inadequately treated. Patients with untreated OSAS are exposed to a significantly higher risk of sudden death, hypertension, stroke, coronary artery disease, congestive heart failure, type 2 diabetes, depression, car accidents, occupational injuries, loss of productivity, and decrease in quality of life [10].

### 1.1. Physiopathology

In OSAS, the disruption of respiratory flow is the result of an obstruction of the upper airways during sleep that can occur during both inhalation and exhalation. The occlusion is formed mainly at the pharyngeal level, which is the only anatomical portion of the respiratory tract without rigid protections and is therefore potentially more susceptible to collapse [4]. In this section of the upper airways, anatomically, the passage of air is ensured by “dilator” muscles that are controlled by the autonomic nervous system and therefore do not require voluntary control [4]. Physiologically, sleep induces a state of relaxation of the body’s muscles, thus also decreasing the activity of the pharyngeal muscles that can no longer maintain the patency of the upper airways, resulting in the collapse of the walls especially at the retropalatal and oropharyngeal level, causing partial (hypopnea) or complete respiratory interruptions (apnea), and then a reduction in the saturation of hemoglobin that normalizes when the episode resolves; in some cases, a gradual increase in PaCO_2_ occurs concomitantly [9].

### 1.2. Clinical Manifestations

Obstructive sleep apnea syndrome is characterized by the presence of night and day signs and symptoms. The nocturnal signs and symptoms are as follows: nocturia; respiratory pauses in sleep; sense of suffocation (choking) during sleep; awakenings at night; snoring at night; night sweating due to increased inspiratory effort; and restless sleep. The diurnal signs and symptoms are the following: excessive daytime sleepiness; reduced memory capacity; difficulty concentrating and paying attention; headache upon waking; tendency to sudden “sleep strokes”; reduction in libido and sexual potency; and changes in mood or behavior [10].

A patient with OSAS also develops cognitive–behavioral changes and mood disorders that cannot be explained by excessive daytime sleepiness alone [5]. Over the past 20 years, numerous studies have tried to determine the extent and causes of ongoing cognitive–behavioral deficits in OSAS, with particular attention to their possible reversibility following treatment of the nocturnal respiratory disorder [2,3,4,5,6,7]. The results, however, are very heterogeneous [8]. The reversibility of cognitive deficits in OSAS following effective treatment of nocturnal respiratory disorder is an open topic of research [9]. The cognitive deficit in OSAS could be a consequence of the deconstruction of sleep, potentially reversible with the restoration of normal hypnic continuity, both of a secondary metabolic damage to hypoxia and intermittent hypercapnia with characteristics of poor reversibility [5]. While the causes of Excessive Daytime Sleepiness (EDS) are more or less directly related to the fragmentation of sleep caused by the frequent arousals associated with apnea, the causes of cognitive–behavioral alterations in OSAS are still to be clarified [7]. At the moment, it is believed that the main causal factors of such alterations are hypnic fragmentation and intermittent nocturnal hypoxia and hypercapnia [7]. In the adult patient, in the absence of cognitive impairment, these do not determine cognitive–behavioral changes, at least in the first years of the disease.

V V Kemstach et al. indicate that obstructive sleep apnea syndrome (OSAS) can have a negative impact on cognitive functioning, primarily executive functions, attention, and episodic memory [11].

The first therapeutic approach in patients with moderate–severe OSAS is represented by the use of C-PAP, but in patients not compliant with treatment, more invasive approaches such as surgery may be considered [12]. Maniaci A. et al. showed that patients with OSAS may benefit from palate surgery, reducing not only the apnea–hypopnea index and daytime sleepiness, but also associated mood comorbidities [3]. Maxillomandibular advancement (MMA) is currently the most effective craniofacial surgical technique for the treatment of obstructive sleep apnea in adults when treatment with C-PAP is ineffective [13]. Shofiq Islam et al. evaluated the reduction in the AHI score after bimaxillary advance: they compared postoperative scores for the apnea–hypopnea index (AHI) and the Epworth sleepiness scale (ESS), and the lowest recorded oxygen saturation between groups after operation; after treatment, the reduction in ESS scores was significant [14].

However, despite a clear literature indicating beneficial effects of positive airway pressure therapy on performance and cognition in OSAS patients, there is a lack of data about surgical procedures such as palate surgery and MMA that address these common complaints of patients [11,12].

We reported a case series of two severe OSAS patients treated with maxillomandibular advancement after C-PAP failure, assessing the variation in cognitive and emotional status after treatment through the use of standardized questionnaires. We decided to investigate the severity of cognitive and depressive symptoms using validated questionnaires including the Beck Depression Inventory (BDI), the Beck Anxiety Inventory (BAI), the Epworth Sleepiness Scale (ESS), and the SF-36 Health Survey [14].

## 2. Case Series

### 2.1. Case 1

A 68-year-old male patient came to our OSAS unit with a severe OSAS diagnosis (AHI of 70 e/h; minimum SpO_2_ 88.9% and ODI 8.6%), a body mass index (BMI) of 29, a neck circumference of 47 cm, and the only comorbidity being arterial hypertension. In addition to daytime sleepiness, the patient presented symptoms related to the humoral and cognitive spheres: irritability, difficulty in concentrating, mood swings, and fatigue. After undergoing a multilevel surgery with tonsillectomy, septoplasty, and uvuloplasty, a new PSG was performed and showed residual disease with AHI of 30/h, so the patient was referred for treatment with C-PAP.

Follow-up after 4 months of use revealed a complete failure of C-PAP due to the lack of adherence by the patient, who felt that the device was extremely invasive, leading to the persistence of daytime sleepiness and cognitive and psycho-emotional symptoms that had already been present before surgical treatment.

In light of the failure of the previous treatments, a drug-induced sleep endoscopy (DISE) was performed to define the most appropriate conduct and highlighted a transversal hypopharyngeal collapse with an increase in posterior airway space (PAS) after a jaw lift maneuver. To evaluate the upper airways volume, the following were performed: RX OPT and Teleradiographs in A-P (posteroanterior cephalometric view) and L-L (lateral cephalometric view). A cephalometric study and cone-beam-computed tomography (CBCT) were also performed to assess the craniofacial pattern.

Based on the results obtained through DISE and cephalometric examinations, the surgical therapeutic solution chosen was MMA.

Six months after the surgery, we performed a control PSG, which showed an almost resolution of the OSAS with an AHI of 13/n with a positional component. The BMI at 6 months after the surgery was 28.

We investigated the cognitive deficits and anxiety–depressive symptoms from the psychological point of view at baseline and after surgery treatment with BDI, BAI, and the SF-36 Health Survey.

The BDI study reported a moderate level of depression in the pre-operatory phase with a score of 28; postoperative rates of depression declined after surgery with a score of 9 points, indicating a significant reduction in depressive symptoms. The BAI administration reported a severe level of anxiety in the pre-operatory phase with a score of 28; postoperative rates of anxiety declined after surgery with a score of 14 points, indicating a significant reduction in anxiety symptoms. The SF-36 administration reported lower levels of physical and mental functioning in the pre-operatory phase compared to postoperative functioning (Table 1).

### 2.2. Case 2

A 23-year-old male subject with severe OSAS, based on an AHI score of 51.8, ODI 8.3, and minimum SpO_2_ 88%, was referred to our maxillofacial surgery unit after two years of C-PAP therapeutic failure. The patient presented a body mass index (BMI) of 32.8, neck circumference of 49 cm, and a daytime sleepiness assessed with the Epworth questionnaire (ESS) with a value of 11. The patient had no comorbidity. In addition to daytime sleepiness, the patient presented symptoms related to the humoral and cognitive spheres: irritability, difficulty in concentrating, mood swings, and fatigue.

To assess the craniofacial pattern through the analysis of the dentoskeletal and soft tissue, a cephalometry, a cone-beam-computed tomography (CBCT), and a DISE were performed.

From the results obtained, a surgical therapeutic intervention of MMA was planned to increase the PAS and reduce the AHI, as well as correct the malocclusion and improve the patient’s aesthetic facial profile. The post-treatment PSG carried out at 6 months revealed a complete and almost total resolution of OSAS with an AHI score of 8.8 events/h and a complete resolution of the symptoms previously reported by the patient, showing a post-surgical ESS value of three and a high degree of satisfaction from both an aesthetic and functional point of view. The BMI at 6 months after the surgery was 25.4.

The BDI study reported a mild level of depression in the pre-operatory phase with a score of 18. Postoperative rates of depression declined after surgery with a score of 9 points, indicating a significant reduction in depressive symptoms.

The BAI administration reported a moderate level of anxiety in the pre-operatory phase with a score of 21. Postoperative rates of anxiety declined after surgery with a score of 10 points, indicating a significant reduction in anxiety symptoms.

The SF-36 administration reported lower levels of physical and mental functioning in the pre-operatory phase compared to postoperative functioning (Table 1).

## 3. Discussion

Daytime sleepiness is considered to be the major and most distinctive symptom of OSAS, determined by the fragmentation of sleep architecture and sleep quality. These mechanisms can also cause impairment of cognitive performance and behavioral and mood disorders. Several studies have shown a correlation between OSAS and an increased risk of depression and anxiety, more frequent and of greater severity in patients with long-lasting apnea, demonstrating a positive linear correlation between AHI, ESS, and ODI indices and mnemonic functions, the extent of cerebrovascular impairment, and behavior [13,14,15,16,17]. In our study, we observed the variation in the cognitive and emotional status of two severe OSAS patients with important age differences (68 y.o. vs. 23 y.o.) but with similar signs of mood and behavioral impairment. In fact, besides showing daytime sleepiness, both patients presented symptoms related to the humoral and cognitive spheres such as irritability, difficulty in concentrating, mood swings, and fatigue. In connection to this, we decided to investigate the severity of cognitive and depressive symptoms using validated questionnaires including the Beck Depression Inventory (BDI), the Beck Anxiety Inventory (BAI), and the SF-36 Health Survey.

To measure the presence of depressive states, we used the Beck Depression Inventory (BDI) [18]. The BDI is a widely used tool for measuring the severity of depressive symptoms. The standard cutoff points for the BDI are as follows: 0–9, indicating minimal depression; 10–18, indicating mild depression; 19–29, indicating moderate depression; and 30–63, indicating severe depression. (Table 2)

The BDI study reported a higher level of depression in the pre-operatory phase in the older patient, with a score of 28, compared to the younger patient, who showed a score of 18. Both patients showed a strong improvement with respect to the rate of depression, which declined after surgery, with a score of 9 points, indicating a significant reduction in depressive symptoms.

The Beck Anxiety Inventory is a self-reported inventory for measuring the severity of anxiety in psychiatric populations [19]. The standard cutoff points for the BAI are as follows: 0–7, indicating minimal anxiety; 8–15, indicating mild anxiety; 16–25, indicating moderate anxiety; and 26–63, indicating severe anxiety. (Table 3) In our patients, the BAI administration reported a severe level of anxiety at least in the older patient, with a score of 28 in the pre-operatory phase, and a moderate level of anxiety, with a score of 21, in the young patient.

Postoperative rates of anxiety declined after surgery for both patients, with a score of 14 and 10 points, respectively, indicating even in this case a significant reduction in anxiety symptoms. Last but not least, to evaluate the quality of life of the patients, we used the SF-36 Health Survey.

The SF-36 was designed for use in clinical practice and research, health policy evaluations, and general population surveys [20]. The SF-36 assesses eight subscales of the health-related quality of life, which are conceptually subsumed in the areas of “physical” and “mental” health. The SF-36 includes one multi-item scale that assesses eight health concepts: (1) limitations in physical activities because of health problems; (2) limitations in social activities because of physical or emotional problems; (3) limitations in usual role activities because of physical health problems; (4) bodily pain; (5) general mental health (psychological distress and well-being); (6) limitations in usual role activities because of emotional problems; (7) vitality (energy and fatigue); and (8) general health perceptions. The scores of each subscale range from 0 to 100 where the higher the score the more favorable the state of health. We observed in both patients that the SF-36 administration reported lower levels of physical and mental functioning in the pre-operatory phase compared to postoperative functioning (Table 1).

Bardwell et al. reported, in 112 patients with OSAS, the severity of depression correlated with age, body mass index, and sleep parameters, probably due to nocturnal hypo-saturation and sleep fragmentation [21].

A growing body of evidence has suggested that the more frequently observed comorbidities of OSAS, such as metabolic imbalance and cardiovascular diseases, could be associated with elevated levels of mental distress [22].

It could be suggested that additional mechanisms noted in OSAS patients, such as inflammation, oxidative stress, or immune system function issues, could be involved in this relationship [23,24,25].

Poor mental health in OSAS patients may be due to the aforementioned comorbidities and their underlying mechanisms. In a similar vein, a recent review highlighted how OSAS affects endothelial dysfunction and neuroinflammation, and that this may initiate or amplify several microvascular and neurovascular pathologic processes that could mediate the association with depression [26].

OSAS patients with depression could experience higher levels of fatigue and lower quality of life than OSAS patients without mood disorders. Depression and anxiety could also make C-PAP therapy difficult. A recent study showed that depression was independently associated with poorer adherence and that a focus on improvement of depressive symptoms could be a strategy to increase the treatment efficacy [26].

Over the past 20 years, maxillomandibular advancement surgery has been widely accepted as the most effective surgical therapy for treating OSAS. Its application, derived from the treatment of facial dysmorphism, was initially based on the observation that abnormalities of the facial skeleton are often detected in patients with OSAS. Such alterations contribute to a reduction in airspace, which is a determining factor in the development of sleep apnea.

Maxillomandibular advancement has been shown to expand pharyngeal and hypopharyngeal airspace by physical expansion of the facial skeletal structure.

The anterior displacement of the maxillomandibular complex allows the tongue to be repositioned anteriorly and tissue tension to be increased, thus reducing the collapse of the velopharyngeal muscles and lateral pharyngeal wall, all significant components in upper respiratory obstruction in the OSAS.

In 2011, the European Respiratory Society published a report, in which it was recognized that the effectiveness of maxillomandibular advancement was comparable to that of CPAP in young subjects without particular excesses of BMI and without pulmonary comorbidities [27].

In the literature are reported different “behavioral patterns” at the basis of the indication to the surgical treatment of OSAS. The maxillomandibular advancement, at first reserved only for dysmorphic patients, is currently also applied to eumorphic patients [27].

The surgical approach is currently indicated in patients with medium–severe OSAS who do not tolerate treatment with C-PAP. Special attention should be paid to obese patients, in which progress, if not accompanied by an adequate weight decrease, is burdened with a high risk of failure or recidivism.

The European Respiratory Society, in a report published in 2011, conducted a review of the literature on the treatment of OSAS in order to identify guidelines that could be universally recognized [27].

In this report, the European Respiratory Society came out in favor of maxillomandibular advancement in selected cases, stating: “maxillomandibular advancement appears to have the same effectiveness as C-PAP in OSA patients who refuse conservative treatment, particularly in a young population without excessive BMI or other comorbidities” [27]. This observation is of great importance in the discussion of OSAS. For the first time, a non-surgical company recognized that the maxillomandibular advancement was superimposed on C-PAP in selected cases.

## 4. Future Directions and Conclusions

Obstructive sleep apnea syndrome is strongly associated with cerebrovascular disorders, and chronic neurodegenerative and inflammatory diseases, leading to a high risk of cognitive impairment in affected patients. However, the relevant literature remains doubtful to date on the efficacy of OSAS treatment on cognitive functions and mood and behavioral impairment. Although several studies have shown that continuous positive airway pressure could improve cognitive domains such as working memory, long-term verbal memory, and short-term visuospatial memory, study designs are often weak, contain a small sample size, and have an inadequate follow-up. Furthermore, insufficient evidence is reported on the efficacy of other available therapeutic approaches such as MAD or OSAS surgery. In our experience, certainly limited by the small number of patients, we described significant improvement in all the analyzed parameters, such as physical and mental functioning, and depression and anxiety rate.

Therefore, to elucidate the role of OSAS therapy on cognitive performance, additional studies are needed, with more evidence to validate the alleged efficacy.

## Figures and Tables

**Table 1:** SF-36 results baseline and after surgery.

SF-36 ITEMS	P1	P2	P1	P2
	BASELINE		AFTER SURGERY	
**PHYSICAL FUNCTIONING**	60	80	70	90
**ROLE LIMITATION DUE TO PHYSICAL PROBLEMS**	50	75	75	95
**BODILY PAIN**	45	75	55	85
**GENERAL HEALTH**	50	80	60	90
**VITALITY**	40	80	60	90
**SOCIAL FUNCTIONING**	50	85	50	90
**ROLE LIMITATION DUE TO PSYCHOLOGICAL PROBLEMS**	40	80	50	90
**MENTAL HEALTH**	40	75	50	85

**Table 2:** Categories for BDI score.

BDI SCORE	CATERGORIES OF DEPRESSION
**0–9**	NONE TO MINIMAL DEPRESSION
**10–18**	MILD TO MODERATE DEPRESSION
**19–29**	MODERATE TO SEVERE DEPRESSION
**30–63**	SEVERE DEPRESSION

**Table 3:** Categories for BAI score.

BAI SCORE	LEVEL OF ANXIETY
**0–7**	MINIMAL
**8–15**	MILD
**16–25**	MODERATE
**26–63**	SEVERE

## Data Availability

Not applicable.
